# Friendship Concept and Community Network Structure among Elementary School and University Students

**DOI:** 10.1371/journal.pone.0164886

**Published:** 2016-10-19

**Authors:** Ana María Hernández-Hernández, Dolores Viga-de Alva, Rodrigo Huerta-Quintanilla, Efrain Canto-Lugo, Hugo Laviada-Molina, Fernanda Molina-Segui

**Affiliations:** 1 Departamento de Física Aplicada. Centro de Investigación y de Estudios Avanzados del Instituto Politécnico Nacional. Mérida, Yucatán, México; 2 Departamento de Ecología Humana. Centro de Investigación y de Estudios Avanzados del Instituto Politécnico Nacional. Mérida, Yucatán, México; 3 Escuela de Ciencias de la Salud-UNEXMAR. Universidad Marista de Mérida. Mérida, Yucatán, México; Semmelweis University, HUNGARY

## Abstract

We use complex network theory to study the differences between the friendship concepts in elementary school and university students. Four friendship networks were identified from surveys. Three of these networks are from elementary schools; two are located in the rural area of Yucatán and the other is in the urban area of Mérida, Yucatán. We analyzed the structure and the communities of these friendship networks and found significant differences among those at the elementary schools compared with those at the university. In elementary schools, the students make friends mainly in the same classroom, but there are also links among different classrooms because of the presence of siblings and relatives in the schools. These kinds of links (sibling-friend or relative-friend) are called, in this work, “mixed links”. The classification of the communities is based on their similarity with the classroom composition. If the community is composed principally of students in different classrooms, the community is classified as heterogeneous. These kinds of communities appear in the elementary school friendship networks mainly because of the presence of relatives and siblings. Once the links between siblings and relatives are removed, the communities resembled the classroom composition. On the other hand, the university students are more selective in choosing friends and therefore, even when they have friends in the same classroom, those communities are quite different to the classroom composition. Also, in the university network, we found heterogeneous communities even when the presence of sibling and relatives is negligible. These differences made up a topological structure quite different at different academic levels. We also found differences in the network characteristics. Once these differences are understood, the topological structure of the friendship network and the communities shaped in an elementary school could be predicted if we know the total number of students and the ties between siblings and relatives. However, at the university, we cannot do the same. This discovery implies that friendship is a dynamic concept that produces several changes in the friendship network structure and the way that people make groups of friends; it provides the opportunity to give analytic support to observational studies. Communities were also studied by gender and we found that when the links among relatives and siblings were removed, the number of communities formed by one gender alone increased. At the university, many communities formed by students of the same gender were also found.

## Introduction

Social scientists have been studying the complex relationships among persons using friendship as a linkage parameter [1–4]. Friendship has a different definition over time; at the preschool level, it is reduced to the relationship among children that play with each other. In adolescence it is defined as the basis of activities that are shared with others, and for adults, the friendship definition rests in face-to-face contact and its frequency [5]. Friendship, as a historical phenomenon, which is complex, dynamic, and sensitive to influence, has been studied in different ways [6, 7].

Social scientists have found that the concept of friendship is not as simple as we may think but depends on several variables [5, 8, 9]. These variables can be described as having five fundamental characteristics [10]: 1) shared activities and/or circumstances, 2) communication and mutual expression, 3) affection or interest in the other, 4) trust and sincerity, and 5) responsibility and mutual commitment.

Friendship, identified as a basis of human and psychological wellbeing [11, 12], is influenced by several environments in which people interact with each other, with their families, and with other social communities such as schools, churches, neighborhoods, etc. It is possible to establish multiple approximations and methodologies that let us understand the friendship phenomenon from biological and natural [13], socioeconomic [1, 14], cultural [3, 15, 16], and psychological perspectives [12, 17–19].

In this study, we adopt an approach that allows us to develop a context for the findings reported concerning friendship, the kind of effects that are produced across a social networks structure, and the communities defined in social network analysis [20–22]. In the literature, there are several studies aimed at identifying the friendship structure in a network that make use of different hypotheses [10, 23–25]. For example, there are university studies where gender was the main issue [26]. In the present manuscript, we address the following question: Is it possible to observe the changes in the concept of friendship through the friendship networks, their properties, and communities?. We analyze four friendship networks, three from elementary schools and one from a university. Two of the three elementary schools are in rural areas. The first two elementary schools (E1 and E2) are public schools located in Temozón South and Abalá, Yucatán, respectively. Those are rural areas where the principal activities are commerce, agriculture, construction, and manufacturing. The population of those areas is small (around 2,000 people), composed mainly of big and close families that live with low or middle incomes. The third elementary school is a public school located in the urban area of Mérida, Yucatán, in a middle-high income zone of the city. The university in which we conducted our study is a private one, located in Mérida, Yucatán. The university has a religious background with traditional values. The students come mostly from families that have middle or high incomes and live in the city. We also were looking for differences in the communities’ composition and topology of the networks according to where the schools are located and the socioeconomic position of those locations.

In this work we use the data presented in the paper entitled *Modeling Social Network Topologies in Elementary Schools* [27]. In that work, the authors conducted a survey of children at three elementary schools, including all the grades from first to sixth, and determined the topological structure of networks that arose from that survey. The age range of the students was from 6 to 12 years. The authors considered friendship as well as enmity networks and found that the topological structures were quite different.

On the other hand, there is a renewed interest in communities and their structure as well as in the algorithms to describe them [28–31]. Computational efficiency is one of the main issues that must be considered when dealing with the problems of finding communities in networks [32, 33]. In this paper we are interested in identifying how communities emerge in the presence of friendships and mixed links. We take into account relationships with siblings and relatives in the students’ network. To do this we employ Newman’s algorithm for all four networks. This algorithm is ideal for finding communities because of its analytical base, which is really clear with respect to network division into communities. We consider this study important because it allows us to provide an analytic and computational basis to observational studies, thus providing the opportunity to engage in interdisciplinary work and open new lines of research for applied physics.

## Methods

We began by analyzing the survey results reported in which three elementary schools were considered [27]. The networks extracted from the three schools: E1, E2, and E3 are made up of 108, 226, and 419 students, respectively. An elementary school is defined as a school for children between 6 to 12 years old. All the elementary schools consisted of six grades, ranging from 1 to 6. A survey at the Universidad Marista located in Mérida city in Yucatán State was also conducted. This fourth friendship network from the university has 1,891 nodes and the ages of the participants are between 18 and 24 years. Specifically, we asked respondents to name his or her friends from the university population. They had to include only close face-to-face friends that did not include friends from electronic social networks (like Facebook, Twitter, etc.). We obtained written permission from the guardians and students enrolled in the study. In all cases, we have written consent from the relevant ethics committees. The study on the elementary schools was approved by the Comité de Bioética para la Investigación en Seres Humanos (COBISH) from Centro de Investigación y de Estudios Avanzados del Instituto Politécnico Nacional. The study at the university was approved by the Comité de Ética e Investigación de la Escuela de Ciencias de la Salud from Universidad Marista de Mérida. It is important to clarify that the surveys, although similar, were not applied in the same form. In the elementary schools, the studies were supervised personally by professionals in the area of human ecology. There was no possibility of using electronic devices because of the remote area the schools are in. In the case of the university, we conducted the survey using the Internet through a platform that we designed for that purpose, after a brief description of the necessary information to do with the research. We were interested in these different academic levels as they represent the initial and final levels of the formal educational system. We choose these particular areas because of the differences between them. In the countryside, the size of the rural communities comprises a population of around 1,000-2,000 people, with big, close families with low-middle incomes. Most of these people have lived there since they were born. On the other hand, Mérida city has a population around 980,000 people, many of them from other parts of the country or even from other nations. The elementary school E3 is located in a zone of the city categorized as middle-high income. This difference in the population size and its composition is interesting with respect to the behavior of the children evaluated, when they are observed from the perspective of a friendship concept evaluation. Even when the structures of the schools are the same (six grades, different classrooms), the number of sibling or relatives is significantly different in the rural areas compared with the urban area of Mérida. This influence of the family ties lets us study the difference in the network owing to those types of links. We chose the university mainly because the difference in age ranges compared with the age ranges in the elementary schools. Table 1 shows all the information concerning the characteristics of the elementary schools and the university. By extracting information from the surveys, we determined the corresponding adjacency matrix (A) for each of the networks. We noticed that the networks were directed, as the adjacency matrix A was not symmetrical.

**Table 1:** Principal characteristics of educational institutions.

Characteristics	E1	E2	E3	University
Number of students (n)	108	226	419	1891
Average students per classroom	18.0	25.1	34.9	24.55
Average number of boys per classroom	9.3	12.0	16.6	11.68
Average number of girls per classroom	8.6	13.1	18.25	12.89
Number of classrooms	6	9	12	77
Location	Rural	Rural	Urban	Urban

Columns E1, E2 and E3 are the labels for each Elementary School. The principal information about the educational institutions and their classrooms are also shown as well.

Friendship should be a reciprocal concept; this means that the networks should be undirected (the adjacency matrix A should be symmetrical), and for this reason all links that were not reciprocal were suppressed [34]. Once all of the A matrices were symmetrical, we started to analyze the networks in terms of their characteristics. The topological information like clustering, density, components, average degree, etc. is shown in Table 2 for all networks. To analyze the influence of links from relatives and siblings (mixed links), we suppressed those links from the original networks (Table 2, and columns E1(NF), E2(NF), and E3(NF)). The next step consisted of choosing an algorithm to obtain the communities for each of the networks. We used the algorithm proposed by Newman in references [35] and [36].

**Table 2:** Principal characteristics of the networks.

	E1	E2	E3	E1 (NF)	E2 (NF)	E3 (NF)	University
Nodes (n)	108	226	419	108	226	419	1891
Links (m)	503	985	1575	298	536	1393	2400
Mixed links	205	449	182	0	0	0	7
Percentage of mixed links	40.75	45.58	11.55	0	0	0	0.29
Connected	yes	yes	yes	yes	no	no	no
Average degree (〈*k*〉)	9.31	8.72	7.52	5.51	4.74	6.64	2.54
Density (*ρ*)	0.087	0.039	0.018	0.056	0.021	0.016	0.0013
Clustering (C)	0.291	0.248	0.226	0.253	0.195	0.255	0.20
Isolated nodes	0	0	0	0	5	2	383
Components	1	1	1	1	1	2	61
Nodes in the Giant Component (n_*g*_)	108	226	419	108	221	415	1313
Links in the Giant Component (m_*g*_)	503	985	1575	298	536	1392	2234
Average degree in the Giant Component (〈*k*_*g*_〉)	9.31	8.72	7.52	5.51	4.85	6.70	3.40

The columns with label (NF) correspond to the Elementary School networks without mixed links.

Newman’s algorithm was implemented in the following way:

1. We obtained the symmetric adjacency matrix with elements *A*_*ij*_.
2. We calculated the modularity matrix *B*:
  $$\begin{array}{c} B_{ij}=A_{ij}-\frac{k_{i}k_{j}}{2m} \end{array}$$ (1)
  where *k*_*i*_ is the degree (number of friends) for each node, and *m* is the total number of links in the network.
3. We determined the eigenvalues and eigenvectors of *B*.
4. We analyzed the eigenvalues of *B* as we had two options:
  - If the largest positive eigenvalue has the largest absolute value, then we continued with step number 5 but otherwise went to (b).
  - If the largest positive eigenvalue was not the largest absolute value, then we recalculated the modularity matrix using the expression
    $$\begin{array}{c} B_{ij}^{new}=B_{ij}-\lambda I \end{array}$$ (2)
    where λ is the eigenvalue with the largest absolute value, *I* is the identity matrix, and *B* is the original modularity matrix. Once we recalculated the modularity matrix, we continued with step 5.
5. Once the largest positive eigenvalue (which has also the largest absolute value) and its eigenvector were obtained, we built the s vector assigning the value −1 or 1 to the entries of this vector depending on the values of the eigenvector. It is −1 if the component of the eigenvector is zero or negative and 1 otherwise.
6. We calculated the modularity Q using the expression
  $$\begin{array}{c} Q=\frac{1}{4m}\sum_{i}\beta_{i}*{\left(v_{i}^{T}\cdot s\right)}^{2} \end{array}$$ (3)
  where *β*_*i*_ is the i-th eigenvalue of the modularity matrix, $v_{i}^{T}$ is its i-th eigenvector, and s is a vector with components 1 or −1.

At this stage of the program, the network is divided into two parts. If the modularity turns out to be larger than zero, then the network can be divided in more parts. If *Q* is less than zero, the network cannot be divided any more. The new partition is implemented with Newman’s algorithm again, calculating the variation of the modularity Δ*Q* in the following way:

1. The modularity matrix was calculated for the subset of nodes that conformed to graph g1
  $$\begin{array}{c} B_{ij}^{\left(g1\right)}=B_{ij}-\delta_{ij}\sum_{k\in g1}B_{ik} \end{array}$$ (4)
  where *g*1 is the first group obtained in the previous division of the network, $B_{ij}^{\left(g1\right)}$ is the modularity matrix associated to g1, *B*_*ij*_ is the matrix of the previous step, and *δ*_*ij*_ is Kronecker’s delta.
2. The eigenvectors and eigenvalues of $B_{ij}^{\left(g1\right)}$ were obtained.
3. Two options are available depending on the eigenvalues:
  - if the largest positive eigenvalue also has the largest absolute value, then the program continues, but
  - if the largest positive eigenvalue was not the one with the largest absolute value, then we recalculated the modularity matrix this time using the expression.
    $$\begin{array}{c} B_{ij}^{new(g1)}=B_{ij}^{\left(g1\right)}-\lambda I \end{array}$$ (5)
  where λ is the eigenvalue with largest absolute value, *I* is the identity matrix and $B_{ij}^{\left(g1\right)}$ is the matrix obtained in step 1.
4. We obtained vector *s*^(*g*1)^ from the eigenvector of the largest eigenvalue assigning −1 to the components that are zero or negative and 1 otherwise. In this way, the −1 components belong to one group and the 1 components to the other.
5. We calculated Δ*Q* defined in the following equation:
  $$\begin{array}{c} \Delta Q=\frac{1}{4m}\sum_{i}\beta_{i}*{\left(v_{i}^{T}\cdot s^{\left(g1\right)}\right)}^{2} \end{array}$$ (6)
  where *β*_*i*_ is the i-th eigenvalue of the modularity matrix *B*^(*g*1)^, $v_{i}^{T}$ is its i-th eigenvector, and *s*^(*g*1)^ is a vector with components 1 or −1.

If Δ*Q* was larger than zero then more partitions in the network can be obtained using the above procedure iteratively. If Δ*Q* was zero or negative for all the subset of nodes of the original matrix, the program stops.

The above algorithm was employed in the elementary schools and university networks. In the elementary schools, we also analyzed the cases where siblings and relatives were not included. To make a comparison of the obtained results, we classified the communities according to two properties: homogeneity and confinement.

- Homogeneity: This property indicates the number of nodes of a community that belong to the same classroom. We say that a community is **homogeneous** if at least 60% of its nodes are students from the same classroom. Otherwise it is **heterogeneous**.
- Confinement: This property indicates the number of students in a classroom belonging to the same community. We say that a community is **confined** if at least 60% of students from a classroom are part of the same community. Otherwise it is **unconfined**.

**Fig 1:**
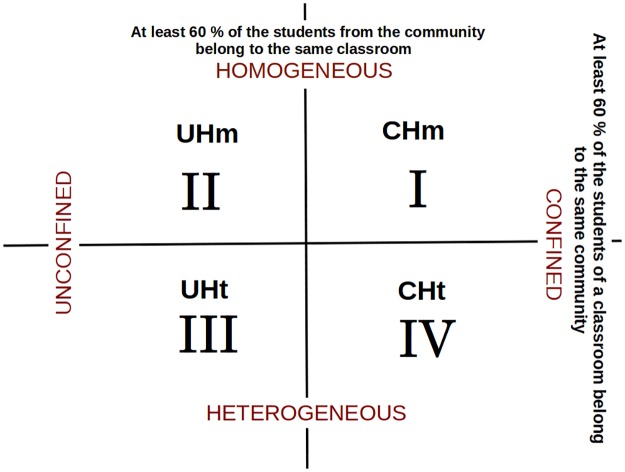
Communities’ classification. In this Cartesian plane we show the classification of the four communities according to the properties of homogeneity and confinement.

Based on these definitions, we classified the communities into four mutually exclusive groups as follow (Fig 1):

1. Confined Homogeneous (CHm).
2. Unconfined Homogeneous (UHm).
3. Unconfined Heterogeneous (UHt).
4. Confined Heterogeneous (CHt).

**Fig 2:**
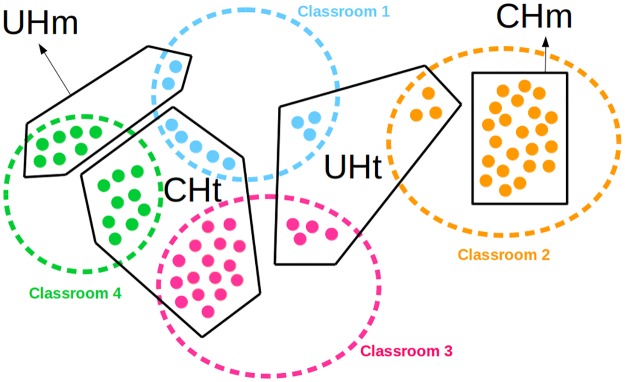
An example of community classification. An example of the way communities are classified depending on the nodal distribution in the classrooms (dashed circles) and communities (polygons in solid black lines). For more detail see the text.

We use Fig 2 to illustrate definitions. In this figure, there are four classrooms numbered from 1 to 4 with 10, 25, 20, and 16 students respectively. There are also four communities: CHm (22 nodes), UHm (9 nodes), UHt (10 nodes) and CHt (30 nodes). The CHm community is integrated with 88% of the students from classroom 2, thus it is confined; the 100% of students in the community are from classroom 2, thus it is homogeneous. The UHm community is composed of 43.7% of students from classroom 4 and 20% of classroom 1, thus it is unconfined; however, 77.7% of the students in the community are from classroom 4, thus it is homogeneous. Community UHt is integrated with 30% of students from classroom 1, 12% of classroom 2, and 20% of classroom 3, thus is unconfined; 30%, of the students in the community are from classroom 1, 30% are from classroom 2, and 40% are from classroom 3, thus is heterogeneous. Finally, the CHt community is composed by 50% of students from classroom 1, 80% of classroom 3, and 56.2% of classroom 4, thus is confined; however, 16.6% of the students in the community are from classroom 1, 53.3% are from classroom 3, and the 30.0% are from classroom 4, thus the community is heterogeneous.

## Results

This study was conducted using the adjacency matrices obtained for each of the networks that are depicted in Table 2. The properties that characterize these social networks are also displayed. The general conditions on which students were at the institutions are shown in Table 1. The three elementary school networks are all connected once the siblings and relatives to the friendship set are incorporated. If we do not consider these links, the networks corresponding to schools 2 and 3 become disconnected. On the other hand, the university network is disconnected, and is formed by 61 components.

We calculate the communities from the largest component of the networks. We start the network analysis by calculating main properties such as the average degree 〈*k*〉, the clustering coefficient *C*, and density *ρ*. We observed that the three elementary schools have large values of 〈*k*〉 even though one of them is in the urban area of Mérida. Moreover, all other characteristics are similar for the elementary school networks.

One interesting thing about the rural schools is that they have a large number of mixed links because of the siblings and relatives that students consider to be friends. In Table 2, we display the information about the mixed links in these networks, where NF means that mixed links were not included. It is worth noticing the existence of a large difference between the two rural schools and the urban school in terms of the mixed links.

**Fig 3:**
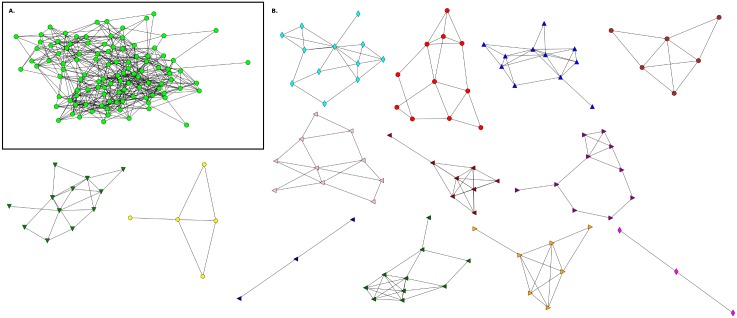
Friendship network for Elementary School E1 and its communities. **A**. This network has n = 108 (nodes), m = 503 (links) and 〈*k*〉 = 9.31. **B**. Communities detected in the network.

**Fig 4:**
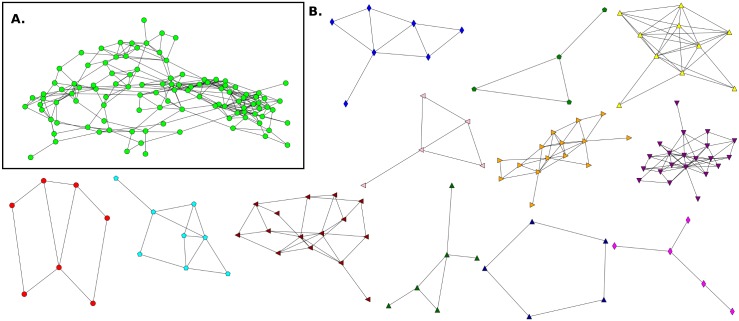
Friendship network for Elementary School and its communities without mixed links E1(NF). **A**. This network has n = 108 (nodes), m = 298 (links) and 〈*k*〉 = 5.51. **B**. Communities detected in the network.

**Fig 5:**
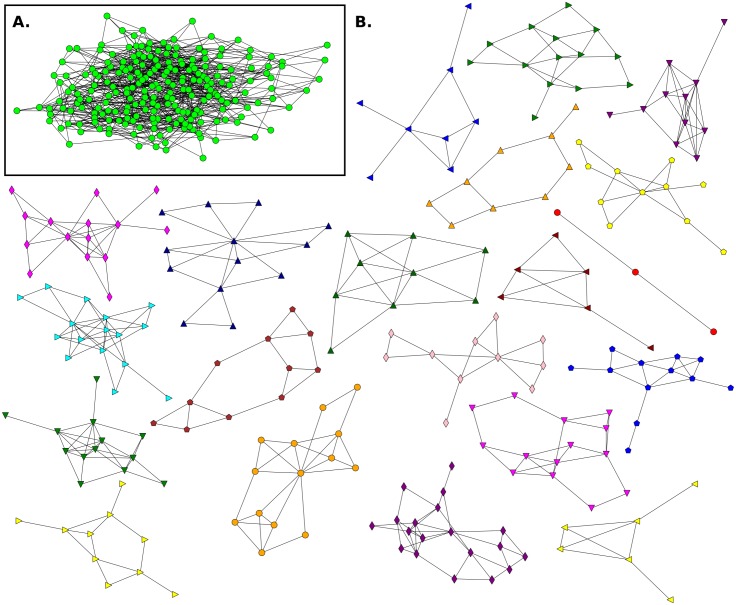
Friendship network for Elementary School E2 and its communities. **A**. This network has n = 226 (nodes), m = 985 (links) and 〈*k*〉 = 8.72. **B**. Communities detected in the network.

**Fig 6:**
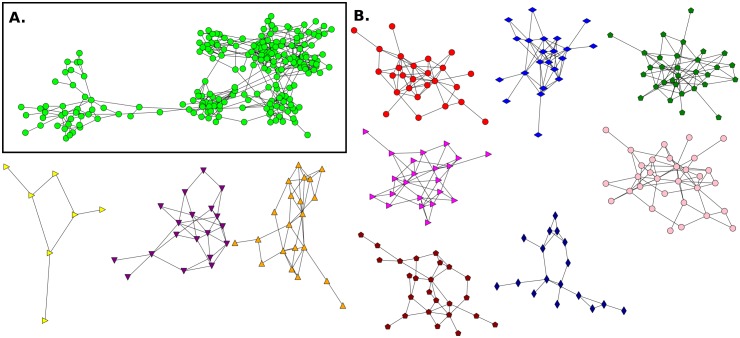
Giant component of Elementary School network and its communities without mixed links E2(NF). **A**. The giant component has n_*g*_ = 221 (nodes), m_*g*_ = 536 (links) and 〈*k*_*g*_〉 = 4.85. **B**. Communities detected in the giant component.

**Fig 7:**
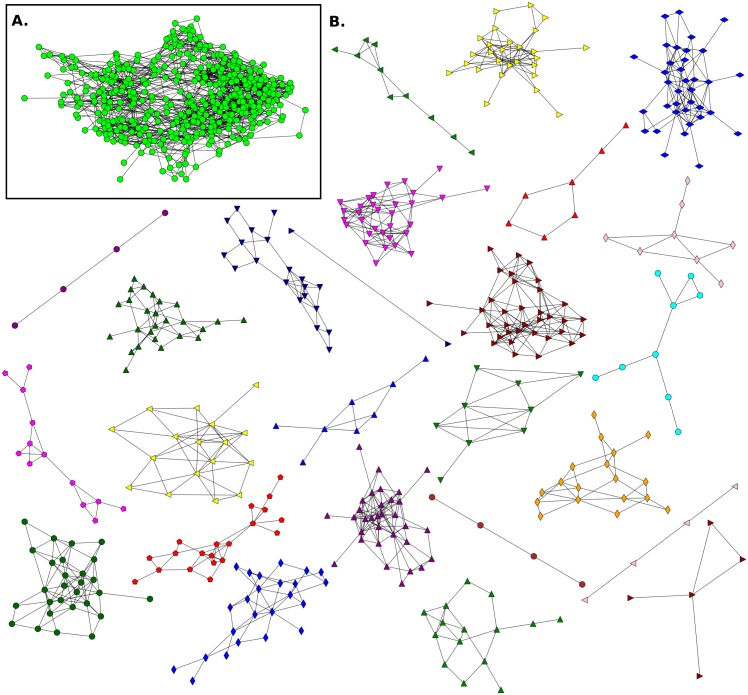
Friendship network for Elementary School E3 and its communities. **A**. This network has n = 419 (nodes), m = 1575 (links) and 〈*k*〉 = 7.52. **B**. Communities detected in the network.

**Fig 8:**
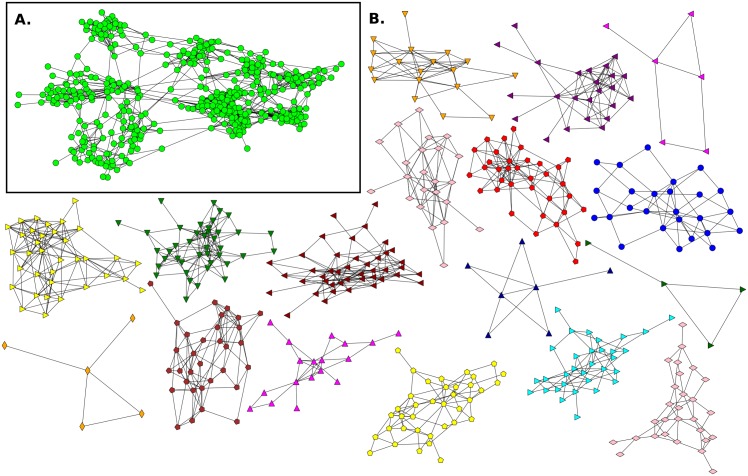
Giant component of Elementary School network and its communities without mixed links E3(NF). **A**. The giant component has n_*g*_ = 415 (nodes), m_*g*_ = 1392 (links) and 〈*k*_*g*_〉 = 6.70. **B**. Communities detected in the network.

**Fig 9:**
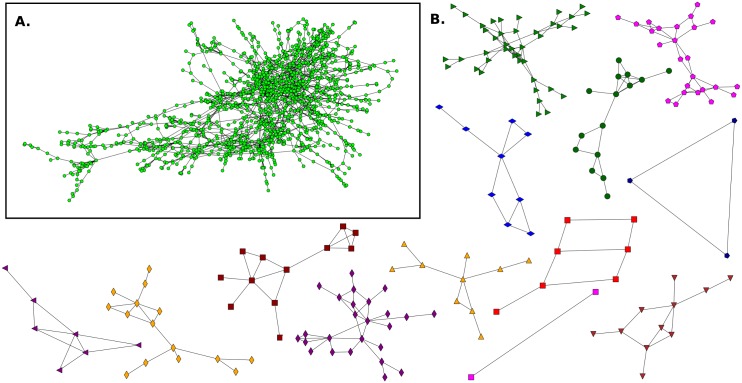
Giant component of University network and its communities. **A**. The giant component has n_*g*_ = 1313 (nodes), m_*g*_ = 2234 (links) and 〈*k*_*g*_〉 = 3.40. **B**. Communities detected in the network.

In rural schools, there are more students who are also considered friends by siblings and relatives. To find the community structure of the networks, we used Newman’s algorithm [35, 36] and investigated the relationship between the friendship concept and that structure. From Figs 3–9 we show the networks and the communities obtained for all schools including the community structure where no mixed links are considered. In Table 3, we give the general characteristics of the networks including some relevant statistics.

**Table 3:** General characteristics of the friendship networks.

	E1	E2	E3	E1 (NF)	E2 (NF)	E3 (NF)	University
Communities	13	20	25	12	10	18	144
N_*p*_ (SD)	8.31(3.10)	11.25(3.68)	16.52(10.65)	9.0(5.07)	22.1(6.68)	24.35(12.49)	10.33(10.46)
*ρ*_*p*_ (SD)	0.50(0.14)	0.36(0.12)	0.32(0.18)	0.45(0.13)	0.19(0.06)	0.27(0.16)	0.53(0.36)
C_*p*_ (SD)	0.46(0.24)	0.41(0.20)	0.27(0.17)	0.37(0.25)	0.21(0.10)	0.33(0.13)	0.19(0.23)
*ρ*_*pp*_ (SD)	0.46(0.15)	0.33(0.13)	0.23(0.20)	0.40(0.20)	0.18(0.06)	0.20(0.17)	0.24(0.46)
*P*_*in*_	0.51	0.52	0.80	0.75	0.86	0.90	0.38
*P*_*out*_	0.49	0.48	0.20	0.25	0.14	0.10	0.62

*N*_*p*_, *ρ*_*p*_ and *C*_*p*_ are the average number of nodes, density, and clustering per community, respectively. *ρ*_*pp*_ is the weighted average density (in respect to the number of nodes). In parentheses, is the standard deviation of every quantity. *P*_*in*_ is the probability to link with someone of the same classroom and *P*_*out*_ is the probability to link with someone from another classroom.

As discussed in the Methods section, the four definitions for the communities give us an interesting piece of information when applied to the networks, displayed in Table 4. We analyzed the component structure of the university network; as mentioned previously, it is formed by 61 components. For the small components we directly applied the community definition, while for the large one (composed by 1,313 nodes), Newman’s algorithm gave us a rich community structure. In the next section, we compare the community structure obtained in this way for the university network to the communities obtained for the elementary schools.

**Table 4:** Communities classification.

	E1	E2	E3	E1 (NF)	E2 (NF)	E3 (NF)	University
Total number of communities	13	20	25	12	10	18	144
CHm communities	0	0	8	2	7	10	1
UHm communities	5	10	7	9	0	8	89
CHt Communities	0	0	0	1	1	0	4
UHt communities	8	10	10	0	2	0	50
Communities composed by boys	2 (UHm)	1 (UHm)	1 (UHm)	3 (UHm)	1 (CHt)	0	18 (17 UHm, 1 UHt)
Communities composed by girls	0	0	0	4 (UHm)	0	4 (UHm)	21 (UHm)

Classification of the communities in the friendship networks.

## Discussion

In Table 2 one can see that the elementary schools networks and the university network have quite different characteristic values, except for the clustering coefficient. The average degree for the university network is considerably lower than the values found for elementary schools. In Table 3, the probabilities of being linked with a partner in the same classroom (*P*_*in*_) and the probabilities of being linked to a partner outside the classroom (*P*_*out*_) are shown. One can observe from these data that the probability of being linked with partners in the same classroom is higher when the mixed links in the network are not included in the elementary schools. On the other hand, in the university network, the probability of being linked to a partner in the same class is lower than the probability of being linked with a partner from another class. That is the reason why 〈*k*〉 and *ρ* are larger for elementary school networks than for university networks. Thus we can say that the concept of friendship creates the topological network in a specific way. We consider the way students are linked in a classroom predictable only in the case when they belong to an elementary school. It is clear that for elementary school students, the friendship concept is so wide that siblings and relatives also belong to the category. One can see in Table 4 that school E3, the one urban school, presents a high number of communities classified as CHm, at least when compared to the other two schools (E1 and E2). It should be noted that it is at this school that the number of relatives and siblings is much fewer than at the other two. This means that friendship is really a spatial concept at this stage of education, where there is a high probability that friends are those within the classroom. To test this hypothesis, we eliminated the mixed links in the schools and checked if there was an increase in the CHm communities. The results are shown in Table 2. When compared with the results depicted in Table 4, we concluded that indeed, the CHm and UHm communities are increased in this case. Therefore, according to the results, spatial confinement favors the formation of friends at this level of education. With university students, it is a different situation. These students have a more defined concept of friendship and therefore do not necessarily consider classmates as friends. One may conclude that spatial confinement has no relevance to having a friend at the university. Even though we also find communities that can be large, most students are in different classrooms; therefore, the type of community is UHt. At this level, we do not have mixed links with relatives and siblings, which is opposite to the case of elementary schools. We have also studied the communities and the gender of the nodes that make up part of each one; at all educational levels, a preference exists to be linked with persons of the same gender, especially when the influence of siblings and relatives is not included (see Table 4). One of the limitations of this type of work is the difficulty of obtaining the data, which is a lot of work and is an slow process that does not provide all the information we need. Another limitation is not having all the information about external factors, which implies the need for not only working with students, but also with their families. There are also regulations such as ethics approvals with which we have to comply. However, we are working to construct a robust database with data not only for friendship networks in Yucatán but also in other places and other grades including secondary school and high school. The strengths of the study lie in exploring the friendship networks and understanding the changes in these at each level and also in learning about the internal variations owing to family ties. Understanding these structures will let us model the dispersion of information or diseases in these kinds of populations. The construction of these kinds of networks is relevant to having a better understanding of the development society. These findings let us know how family ties could affect other areas of life, and are a step to understanding the complexity of social interactions at different ages. In Yucatán, children are in the principal groupaffected by diseases such as Chikungunya or the Zika virus, influenza or dengue fever. This knowledge could provide the opportunity to create another kind of work in future, for example, how to model the dispersion of illness in these types of social communities once we know the topology of the network and how children form communities in the network. This work is one step in constructing a robust database to start with directional work into diseases predict not only in Yucatan but also in other areas with similar problems.

## Conclusion

From the study of friendship networks presented we can conclude that, in general, the differences in topologies between the elementary school networks and university networks are an effect attributable to the concept of friendship, which changes over a lifetime. However, a bigger effect is visible when communities in the networks are studied as the basis of family ties, friendship ties, and spatial confinement (Tables 2, 3, and 4). We can also conclude, on the basis of the networks analysis that: 1) the concept of friendship is clearly different at both educational levels and this can be observed from the topology characteristics of the networks and the difference in the percentage of mixed links present in the networks, 2) the mixed links produce a social effect in the elementary schools, which changes the distribution of students in the communities; however, the presence of relatives and siblings does not eradicate completely the effect produced by spatial confinement, 3) it could be possible to predict the topology of other elementary schools if we knew the number of students and mixed links between them; however, this is not possible at the university, 4) at the university, a clear difference exists between the kinds of relationships in which the students are involved, and that is why the percentage of mixed links in this network is low, and 5) it is apparent that the friendship concept has a high personal component that produces a low reciprocity in the university network, a disconnected network, and a large number of small components.

## Supporting Information

S1 Fig Copies of the questionnaires used in the surveys. (PDF)

S1 Dataset Adjacency Matrix. Copies of the seven adjacency matrices used in the study.
(ZIP)

S1 File Ethic letters and guardians’ permission letters. (ZIP)
