# Supplementary material for: Friendship Concept and Community Network Structure among Elementary School and University Students
Source: PLoS One. 2016 Oct 19;11(10):e0164886. doi: 10.1371/journal.pone.0164886 (PMC5070781; doi:10.1371/journal.pone.0164886)
Supplement: S1 Fig — (PDF) [file pone.0164886.s001.pdf]

CENTRO DE INVESTIGACIÓN Y DE ESTUDIOS AVANZADOS DEL IPN, UNIDAD MÉRIDA.
DEPARTAMENTOS DE ECOLOGÍA HUMANA Y FÍSICA APLICADA

CUESTIONARIO DE PREGUNTAS ABIERTAS SOBRE INTERACCIONES ENTRE ALUMNOS DE LA ESCUELA PRIMARIA DEL MUNICIPIO DE TEMOZÓN SUR DE ABALÁ, EN YUCATÁN

Esto no es un examen. Estamos haciendo un estudio sobre las relaciones entre los niños de la escuela. Nos interesa conocer tu opinión. Por favor, contéstanos las siguientes preguntas y si tienes duda consúltanos. Gracias por tu apoyo.

1. Fecha (día, mes, año) _______/______/______
2. Nombre (sólo nombres) ____
3. Edad ________ 4. Sexo: Mujer ( ) Hombre ( )
5. ¿Dónde naciste? ____
6- ¿Cuantos hermanos tienes? ______

7- De la lista que se te da, señala quienes son tus mejores amigos [√].

8- Escribe el nombre y grado de tus mejores amigos, que estudian en esta escuela, pero son de otro grado.

9- De la lista que se te da, señala con quienes no te llevas bien [X].

10- Escribe el nombre y grado de los alumnos con los que no te llevas bien, que estudian en esta escuela, pero son de otro grado.

11- Si tienes hermanos o hermanas (primos o primas) estudiando en esta escuela, escribe sus nombres, escribe también de que grado es cada uno.

12- ¿Cómo son tus compañeros de clase?
[ ] Algunos molestan mucho
[ ] Peleamos mucho
[ ] Somos buenos amigos

Lista de alumnos de Segundo año

1. Nombre Apellido [ ]
2. Nombre Apellido [ ]
3. Nombre Apellido [ ]
4. Nombre Apellido [ ]
5. Nombre Apellido [ ]
6. Nombre Apellido [ ]
7. Nombre Apellido [ ]
8. Nombre Apellido [ ]
9. Nombre Apellido [ ]
10. Nombre Apellido [ ]
11. Nombre Apellido [ ]
12. Nombre Apellido [ ]
13. Nombre Apellido [ ]
14. Nombre Apellido [ ]
15. Nombre Apellido [ ]
16. Nombre Apellido [ ]
17. Nombre Apellido [ ]
18. Nombre Apellido [ ]
19. Nombre Apellido [ ]
20. Nombre Apellido [ ]

CENTRO DE INVESTIGACIÓN Y DE ESTUDIOS AVANZADOS DEL IPN, UNIDAD MÉRIDA.

HUMAN ECOLOGY AND APPLIED PHYSICS DEPARTMENTS

OPEN QUESTION SURVEY ABOUT SOCIAL INTERACTIONS IN PRIMARY SCHOOL STUDENTS IN TEMOZÓN, SOUTH ABALÁ, YUCATÁN

This is not an exam. We are doing an study about relationships among children in the school. We are interested to know your opinion. Please answer the next questions and if you have any doubt please ask to us. Thank you for your help and suport.

1. Date (day, mounth, year) _______/______/______
2. Name (just given name) ____
3. Age ________ 4. Gender: Female ( ) Male ( )
5. ¿Where were you born?____
6- ¿How many brother and sisters do you have? ______
7- From the list you have, choose your best friends [√].
8- Write the name and grade of your best friends inside the school, but in another classroom.

9- From the list you have, choose who you do not get along with [X].

10- Write the name and grade of student that you do not get along with inside the school, but in another classroom.

11- If you have brothers or sisters (or cousings) studying in your school, write their given names and the classroom of each one of them.

12- ¿ How do your classmates behave?
[ ] Some of them are very anoying
[ ] We fight a lot
[ ] We are good friends

Students list from second grade

1. First name Middle name [ ]
2. First name Middle name [ ]
3. First name Middle name [ ]
4. First name Middle name [ ]
5. First name Middle name [ ]
6. First name Middle name [ ]
7. First name Middle name [ ]
8. First name Middle name [ ]
9. First name Middle name [ ]
10. First name Middle name [ ]
11. First name Middle name [ ]
12. First name Middle name [ ]
13. First name Middle name [ ]

Cinvestav
UNIDAD MERIDA

Estudio de la Variación de Masa Corporal y Balance Energético Inducido Socialmente

MARISTA

ENCUESTA PARA ESTABLECER LOS ENLACES DE AMISTAD ENTRE LOS ESTUDIANTES DE LA UNIVERSIDAD MARISTA DE MÉRIDA:

De manera anticipada le agredecemos por su colaboración y su tiempo. Le recordamos que toda la información que usted nos suministre por este medio será tratada de manera confidencial, anónima, y no será empleada para otro fin que el de la investigación científica. Todos los datos suministrados por usted serán tratados de acuerdo a las normas establecidas por el Instituto Federal de Acceso a la Información y Protección de Datos. También le exortamos a llenar el siguiente formulario con la mayor sinceridad posible y le agradecemos de nuevo por toda su colaboración y su disposición.

Por favor indique su número de matricula

Los siguientes datos que le pedimos por favor registrar, son datos de sus amigos pertenecientes a la comunidad de estudiantes de la Universidad Marista de la ciudad de Mérida Yucatán. Le pediremos por favor elija dentro de los menús desplegables los nombres de sus amigos más cercanos (es decir con aquellos con los que comparte más tiempo haciendo actividades como estudiar, hacer algún deporte, o alguna actividad que implique pasar tiempo cara a cara con esa persona). Recuerde por favor que toda esta información es solo recabada para fines científicos y solo el personal del grupo investigación tendra acceso a ella para fines de estudios estadísticos.

Siguiente

Estudio de la Variación de Masa Corporal y Balance Energético Inducido Socialmente

Cinvestav UNIDAD MERIDA

MARISTA

Por favor lea las siguientes instruciones y sigalas con cuidado

Por favor seleccione en las siguientes listas desplegables la carrera, el semestre y el nombre de sus amigos y amigas pertenecientes a la población estudiantil de la Universidad Marista. Recuerde por favor que la definición de amigos más cercanos para nuestro estudio se refiere a aquellos con los que realiza más actividades cara a cara, como estudiar, trabajar, practicar un deporte, etc; ó con quienes se ve más seguido para comer, tomar un café etc.Si desea borrar alguno de los nombres ingresados, elija en el menú de carreras la opción "-Borrar dato-" y en el menú de semestre elija la opción "0". Cuando termine de ingresar los datos, por favor de click en el botón cuyo texto dice "Siguiente".

	Carrera	Semestre	Nombre
a.	---Seleccione una carrera---	semestre	Seleccione el nombre de su amigo/a

Agregar amigo/a

Estudio de la Variación de Masa Corporal y Balance Energético Inducido Socialmente

Para finalizar la recolección de los datos que usted muy amablemente nos ha suministrado, le pedimos por último escriba su nombre en el cuadro que aparece en la parte inferior de esta página. Esta, será su autorización para que guardemos sus datos y los usemos única y exclusivamente para fines de investigación científica. Le pedimos también lea con atención y si esta usted de acuerdo, escriba su nombre completo y haga click en el boton Guardar al final de esta página. De lo contrario, si no desea que hagamos uso de sus datos, por favor de click en el boton Declinar al final de la página.

Por este medio doy autorización al Centro de Investigación y de Estudios Avanzados del Instituto Politécnico Nacional CINVESTAV-IPN Unidad Mérida y a la Universidad Marista de Mérida para recopilar todos mis datos personales suministrados por mí en persona a través de este medio electrónico. Tengo conocimiento de que las dos instituciones anteriormente mencionadas, llevan a cabo un proyecto de investigación científica para el cual mis datos son necesarios para fines estadísticos. Así mismo se me ha informado que el tratamiendo de mis datos se hará de manera anónima y serán resguardados conforme a lo estipulado por el Instituto Federal de Acceso a la Información y Protección de Datos. Doy mi autorización teniendo conocimiento del fin por el cual han sido recabados.

Su nombre completo: ____________________

GUARDAR ---Declinar---

Body Mass Variation and Energy Balance Study.
The Social Influence Contribution

Cinvestav UNIDAD MERIDA

MARISTA

Please read carefuly all the instructions to response de survey properly.

SURVEY TO STABLISH THE FRIENDSHIP NETWORK IN THE MARISTA UNIVERSITY, MÉRIDA YUCATÁN.

We appreciate your help and your time. We remind you that all the information that you gave us through this media will be treated in a confidential and anonymous way and only will be used for scientific purposes.

Please, introduce your student id number [ ]

In the next page, we ask you to give us, using our student list, the names of your most close friends in the university. (it means, the students you get along with and share time and activities as practice a sport, go to study, go to eat, drink a coffee, go out to a party, or any other activity that implies to share time in a face to face interaction). Please remember that all the information that you gave us is going to be used only for scientific purposes and only the researchers in the interdisciplinary group would have access to the information for statistical study.

Next

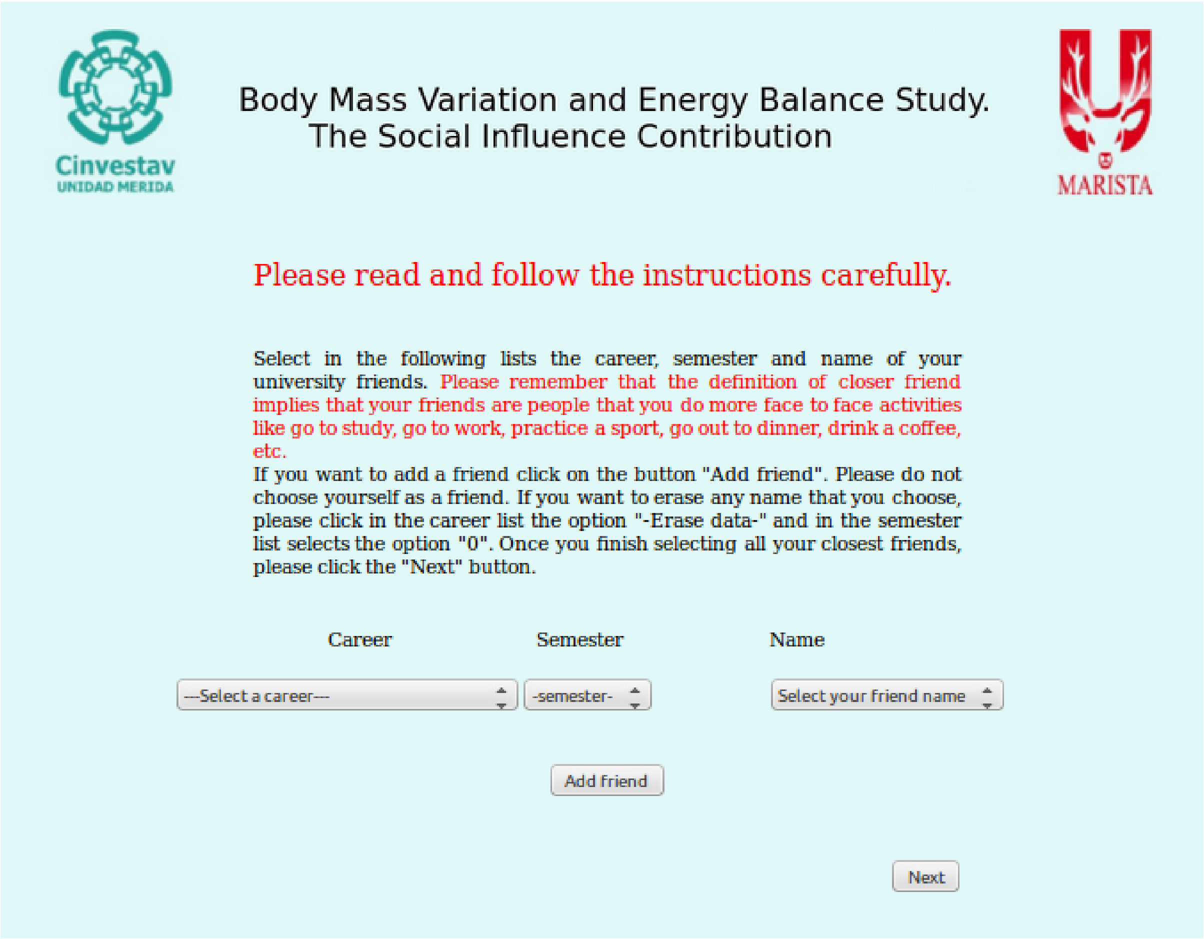
Cinvestav
UNIDAD MERIDA
Body Mass Variation and Energy Balance Study.
The Social Influence Contribution
MARISTA
Please read and follow the instructions carefully.
Select in the following lists the career, semester and name of your university friends. Please remember that the definition of closer friend implies that your friends are people that you do more face to face activities like go to study, go to work, practice a sport, go out to dinner, drink a coffee, etc.
If you want to add a friend click on the button "Add friend". Please do not choose yourself as a friend. If you want to erase any name that you choose, please click in the career list the option "-Erase data-" and in the semester list selects the option "0". Once you finish selecting all your closest friends, please click the "Next" button.
Career
Semester
Name
---Select a career---
-semester-
Select your friend name
Add friend
Next

Body Mass Variation and Energy Balance Study.
The Social Influence Contribution

To finish the survey, we ask you to please read the next text and if you accept please write your full name in the white box on the bottom of this page and click on the button "AGREE". If you do not want to share with us the information that your previously give on the previous page, click the button "---I do not agree---".

Using this media I give my authorization to the Centro de Investigacion y de Estudios Avanzados del Instituto Politécnico Nacional CINVESTAV-IPN Unidad Mérida and the Marista University of Mérida to save and use all the data that I give them through this web platform. I know that both institutions, previously mentioned, are doing a research study and my data are needed to the success of the research. I had been previously informed about the treatment of my data, that would be treated anonymously and according to the lineaments established by the Federal Institution for Information Access and Protection. I give my authorization knowing the purposes of the research study and the form that all my information is going to be used.

Your full name:

AGREE

---I do not agree---
