# Supplementary material for: Friendship Concept and Community Network Structure among Elementary School and University Students
Source: PLoS One. 2016 Oct 19;11(10):e0164886. doi: 10.1371/journal.pone.0164886 (PMC5070781; doi:10.1371/journal.pone.0164886)
Supplement: S1 File — (ZIP) [file pone.0164886.s003.zip › E1.pdf]

CENTRO DE INVESTIGACION Y DE ESTUDIOS AVANZADOS DEL I.P.N. UNIDAD MERIDA
DEPARTAMENTO DE ECOLOGIA HUMANA
Y FÍSICA APLICADA

Cinvestav

Temozón Sur de Abalá, Yucatán, Enero 24 de 2011

ASUNTO: AUTORIZACIÓN PARA APLICAR CUESTIONARIO

SOBRE REDES SOCIALES EN NIÑOS DE LA ESCUELA PRIMARIA

Profesor Daniel Ambrosio Mejía
Director de la Escuela Primaria "Silvestre Chi"

Somos un grupo de investigadores que trabajamos en el Departamento de Física Aplicada y de Ecología Humana del Centro de Investigación y de Estudios Avanzados del Instituto Politécnico Nacional, Unidad Mérida, realizando un estudio sobre educación ambiental y la forma en que se dispersa la cultura ambiental en la escuela y comunidad.

El propósito de esta carta es solicitarle su autorización para que los alumnos de su escuela participen en este estudio. El objetivo de su participación consiste en conocer cuáles son las redes de interacción que establecen los niños con familiares, otros niños y niñas de la escuela. Para obtener esta información requerimos aplicar un cuestionario de 12 preguntas.

Con la información obtenida y procesada se modelarán las redes sociales de relación en la escuela, mismas que permitirán aterrizar la forma en que se da la dispersión de la cultura ambiental, propagación de enfermedades, etc. La información proporcionada por los niños y los participantes en este proyecto de investigación será estrictamente confidencial y usada con fines académicos para la elaboración de reportes e informes del CINVESTAV y publicación de artículos en revistas especializadas.

Dirección: Carretera Antigua a Progreso Km. 6 C.P. 97310 Mérida, Yucatán Dirección Postal A.P. 73 "Cordemex" Yucatán Tels. (999) 942-94-00 ext. 2533 Fax (999) 981 46 70 Email: dviga@mda.cinvestav.mx
ww.mda.cinvestav.mx

Agradecemos de antemano su colaboración para que los alumnos de su escuela participen en esta encuesta y quedamos a sus órdenes para cualquier información que se requiera.

A T E N T A M E N T E

Dr. Rodrigo Huerta Quintanilla
Responsable Académico del Proyecto

Dra. María Dolores Viga de Alva
Auxiliar de Investigación

M. C. Efraín Canto Lugo
Auxiliar de Investigación

Dirección: Carretera Antigua a Progreso Km. 6 C.P. 97310 Mérida, Yucatán Dirección Postal A.P. 73 "Cordemex" Yucatán Tels. (999) 942-94-00 ext. 2533 Fax (999) 981 46 70 Email: dviga@mda.cinvestav.mx
ww.mda.cinvestav.mx

Cinvestav-Mérida

Departamento de Física Aplicada

CINVESTAV

A quien corresponda:

Carta de consentimiento del director

Fecha: 24 de enero de 2011

Título: Modelando redes sociales en escuelas primarias

He leído y entendido en mi lengua materna el documento sobre la descripción de la investigación.

Estoy de acuerdo en participar en este proyecto de investigación conforme a lo descrito en el documento que describe dicho proyecto.

Se me ha proporcionado una copia de ese documento así como de la carta de consentimiento para mi archivo.

Los investigadores han acordado no revelar la identidad ni datos personales de los participantes si la información resultado de este proyecto es presentada o publicada en cualquier formato público.

Nombre de la persona que da el consentimiento Daniel Ambrocio Mejía

Firma..

Centro de Investigación y de Estudios Avanzados del Instituto Politécnico Nacional – Unidad Mérida
Carretera Antigua a Progreso Km. 6, A.P. 73 Cordemex 97310, Mérida, Yucatán, México
Tel. +52 (999) 942-94-78, Fax. +52 (999) 981-29-17, e-mail: infofis@mda.cinvestav.mx
www.mda.cinvestav.mx
