# Supplementary material for: Friendship Concept and Community Network Structure among Elementary School and University Students
Source: PLoS One. 2016 Oct 19;11(10):e0164886. doi: 10.1371/journal.pone.0164886 (PMC5070781; doi:10.1371/journal.pone.0164886)
Supplement: S1 File — (ZIP) [file pone.0164886.s003.zip › University.pdf]

MARISTAS

Universidad Marista de Mérida

Mérida, Yucatán a 1 de Septiembre de 2015

M en C. Ana María Hernández Hernández

Investigadora Responsable

Y A QUIEN CORRESPONDA:

Por este conducto comunico que el estudio **"Estudio de la variación de masa corporal y balance energético inducido socialmente"** fue sometido en su momento al Comité de Etica e Investigación de la Escuela de Ciencias de la Salud para su Dictamen. Los responsables: la estudiante de doctorado Ana María Hernández Hernández, así como el Dr. Hugo Laviada Molina, el Dr. Rodrigo Huerta, y la M en C Fernanda Molina Seguí comunican que se trata de un estudio que incluye encuestas y mediciones antropométricas no invasivas, que no implican un riesgo para la salud.

Dr. Marco Antonio Escalante Rodriguez
Presidente del Comité de Etica e Investigación

D Periférico Norte Tablaje Catastral 13941 Carretera Mérida-Progreso. Mérida, Yucatán
T (999) 942 97 00
W www.marista.edu.mx

Universidad Marista
