# Supplementary figures and images for: Friendship Concept and Community Network Structure among Elementary School and University Students
Source: PLoS One. 2016 Oct 19;11(10):e0164886. doi: 10.1371/journal.pone.0164886 (PMC5070781; doi:10.1371/journal.pone.0164886)

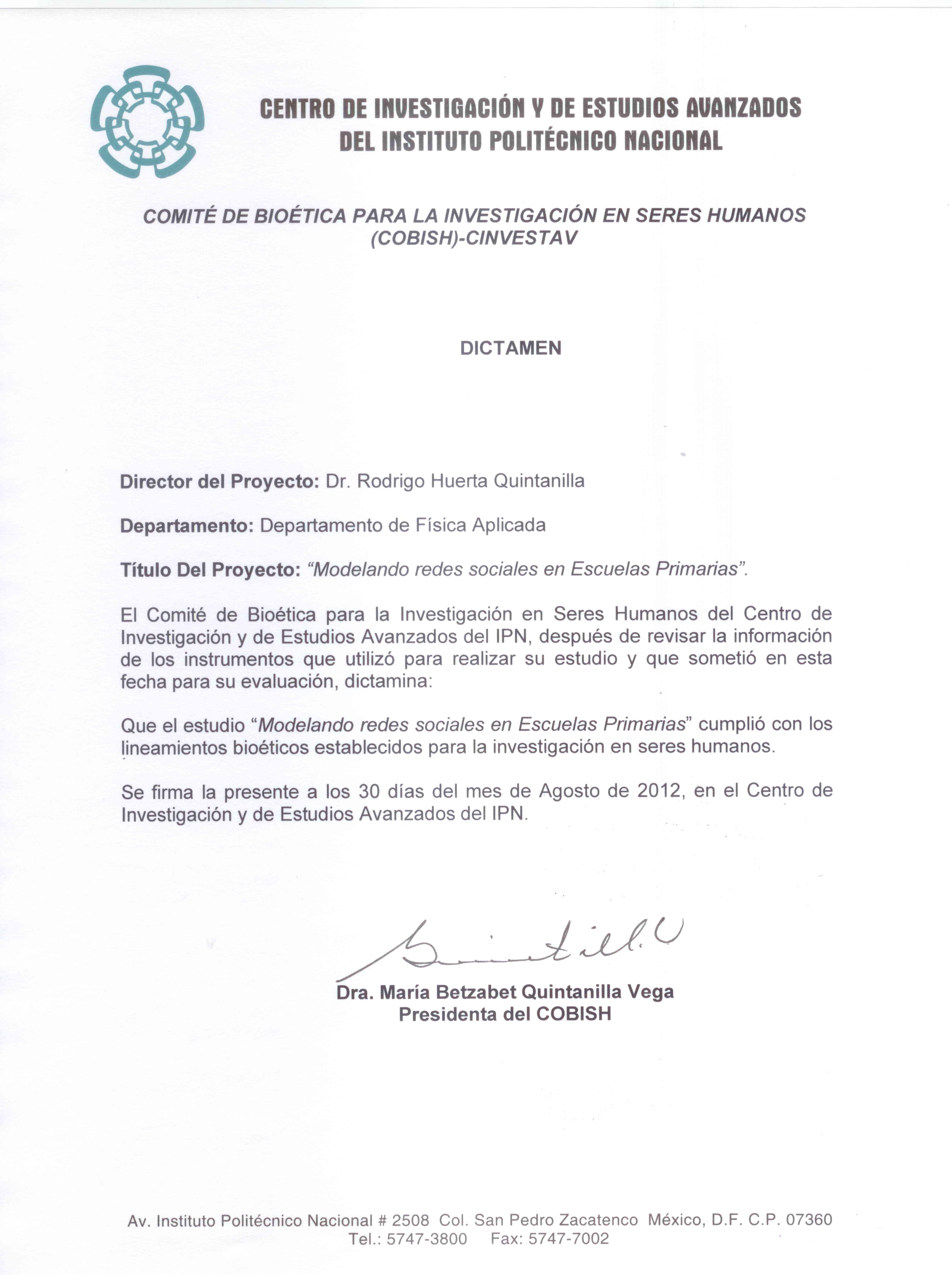

Supplement: S1 File — (ZIP) [file pone.0164886.s003.zip › COBISH Dictamen.jpeg]
